# The Multifactorial Origin of Respiratory Morbidity in Patients Surviving Neonatal Repair of Esophageal Atresia

**DOI:** 10.3389/fped.2014.00039

**Published:** 2014-05-05

**Authors:** Ana Catarina Fragoso, Juan A. Tovar

**Affiliations:** ^1^INGEMM and Idipaz Research Laboratory, Department of Pediatric Surgery, Hospital Universitario La Paz, Madrid, Spain; ^2^Department of Pediatrics, Universidad Autonoma de Madrid, Madrid, Spain; ^3^Faculty of Medicine, University of Porto, Porto, Portugal

**Keywords:** esophageal atresia, tracheoesophageal fistula, respiratory tract disease, lung, development, human, rodent models

## Abstract

Esophageal atresia with or without tracheoesophageal fistula (EA ± TEF) occurs in 1 out of every 3000 births. Current survival approaches 95%, and research is therefore focused on morbidity and health-related quality of life issues. Up to 50% of neonates with EA ± TEF have one or more additional malformations including those of the respiratory tract that occur in a relatively high proportion of them and particularly of those with vertebral, anal, cardiac, tracheoesophageal, renal, and limb association. Additionally, a significant proportion of survivors suffer abnormal pulmonary function and chronic respiratory tract disease. The present review summarizes the current knowledge about the nature of these symptoms in patients treated for EA ± TEF, and explores the hypothesis that disturbed development and maturation of the respiratory tract could contribute to their pathogenesis.

## Introduction

Progress in surgical techniques and perinatal care allowed dramatic improvement of survival in the decades elapsed since the first successful primary repair of esophageal atresia with tracheoesophageal fistula (EA ± TEF) in 1941 (1). However, even after an uneventful postoperative course, a large proportion of these children suffer long-standing respiratory tract disease. This is, in fact, as frequent in EA ± TEF survivors as in children operated upon for congenital diaphragmatic hernia, a condition in which lung hypoplasia and pulmonary hypertension are frequent (2). The persistent respiratory symptoms found in children with EA ± TEF are recurrent bronchitis, brassy cough, chronic cough, repeated pneumonia, and asthma-like wheezing. Although some of them may be accounted for by documented tracheomalacia, esophageal dysmotility, gastroesophageal reflux (GER), or surgical complications, up to 75% of survivors still have abnormal pulmonary function (obstructive/restrictive) apparently not related to these abovementioned conditions (3, 4). Although respiratory morbidity tends to improve with age, the chronic cough, bronchial constriction and hyper-responsiveness can persist or even become more frequent in adulthood (5–7).

The common origin of both the digestive and respiratory systems from the embryonic foregut explains why abnormal regulation of its development causes malformations of both systems. Accumulating evidence suggests that lung lesions acquired during early development can lead to persistent structural damage and respiratory function impairment during postnatal life. The present review examines the link between disturbed lung development during embryogenesis of EA ± TEF and the respiratory morbidity in patients with this malformation.

## Respiratory Tract Disease in EA ± TEF Survivors

Patients with EA ± TEF suffer respiratory symptoms more often and more persistently than other individuals. In the long term, they are reported in up to 72% of surviving adolescents and adults (4, 8, 9). The most frequent symptoms are chronic (ranging from 8 to 86%) and barky cough (24–75%), bronchitis (14–74%), dyspnea on exertion or poor tolerance to exercise (19–37%), and pneumonia (34–48%) (3, 4, 8–14). Doctor-diagnosed asthma, atopy, and bronchial hyper-responsiveness also appear to be quite frequent with prevalences ranging from 16 to 65% (4, 8–15). In fact, atopy is responsible for a significant proportion of respiratory morbidity in older patients whose inflammatory profile based on bronchial biopsies and exhaled nitric oxide differs from the typical asthma patients (16).

Abnormal pulmonary function tests are demonstrated in up to 96% of children, adolescents, and adults previously treated for EA ± TEF (14). The restrictive (35–57%) pattern prevails over the obstructive (11–55%) or mixed ones (3, 4, 8). Legrand et al. reported abnormal spirometry not related to prematurity; intrauterine growth retardation; associated anomalies; vertebral, anal, cardiac, tracheoesophageal, renal, limb (VACTERL) association; esophageal dilations; gastrostomy; fundoplication; and GER or dysphagia in 75% of EA ± TEF survivors (3). Peetsold et al. also found that restrictive impairments were similarly frequent in EA ± TEF patients irrespective of the need for anti-reflux surgery (13).

Although accumulating data suggest that respiratory symptoms in these patients improve with age, their quality of life is significantly impaired for these reasons when they become adults (4). In fact, chronic cough and wheezing do not improve with age and in a subset of patients even become more frequent (4–7). Lilja et al. in a large study with 125 EA ± TEF survivors ranging from 1 to 20 years of age reported that shortness of breath (53%) and respiratory infections (40%) were more frequent in the group aged 16–20 than in the groups aged 1–5 (36 and 11%, respectively), 6–10 (21 and 25%, respectively), and 11–15 (38 and 56%, respectively); other frequent symptoms such as coughing, impaired exercise capacity, and asthma medication had a similar or slightly reduced prevalence in the oldest group (1–5 years group: 43, 27, and 35% vs. 16–20 years groups: 36, 20, and 36%, respectively) (7).

Finally, respiratory complications are major causes of early death in EA ± TEF patients immediately following cardiac malformations and chromosomal anomalies, and are even the main reasonable responsible for late deaths (the majority occurring during the first year of life) (17–19).

## Epidemiology of EA ± TEF and Associated Respiratory Malformations

EA ± TEF occurs in 1 per 3000 live births. It is two to three times more common in twins, and although most cases are sporadic, as many as 10% are associated with chromosomal anomalies (20). Trisomies and several syndromes and associations have EA ± TEF as a feature such as anophthalmia–esophageal–genital (AEG); coloboma; heart anomaly; choanal atresia, retardation, genital, and ear (CHARGE); Feingold or X-linked Opitz syndromes; and the most commonly related VACTERL association. The spectrum of the malformation comprises five anatomical subtypes: EA with distal TEF (86%) followed by isolated EA without TEF (7%), TEF without EA (4%), EA with proximal TEF (2%), and EA with proximal and distal TEF (<1%) (20).

Approximately 50% of neonates with EA ± TEF have one or more additional malformations: cardiovascular (29%), anal (14%), genitourinary (14%), gastrointestinal (13%), or skeletal (10%) (20). Respiratory malformations are present in 6% of children with EA ± TEF (and in 13% of those who came to necropsy). These proportions go up to 47% in patients with the VACTERL association (20–22). Hypoplasia, fusion (horseshoe) or agenesis of the lung (23–29), and structural, innervatory, and epithelial differentiation anomalies of the tracheobronchial tree have been described in this condition (22, 30–33).

## Mechanisms of EA ± TEF

The etiology of EA ± TEF is largely unknown. Isolated EA ± TEF is probably caused by an insult that occurs during the narrow gestational window of tracheoesophageal cleavage. Very little is known about its causes. The lack of a heritable pattern and the evidence that environmental agents acting *in utero* in human individuals such as maternal alcohol use, phenylketonuria, diabetes, and metamizole or intrauterine exposure to adriamycin and levothyroxine in rodents support a multifactorial origin (34–36). However, in cases of syndromic EA ± TEF, trisomies and other chromosomal anomalies as well as several genetic pathways involving genes such as *N-MYC, SOX2*, and *CDH7* have been shown to contribute to the pathogenesis (37).

The precise mechanisms of tracheoesophageal malformations are still unclear. Nevertheless, the recent development of animal models that closely mimics the human phenotype allowed significant advances in understanding of the dysmorphogenetic processes.

During the fourth week of embryonic life in humans and around day 12 in rats, the foregut endoderm differentiates into a ventral respiratory part and a dorsal esophageal part. After emergence of the tracheobronchial bud from the floor of the foregut, the respiratory and esophageal parts of the foregut begin to separate. The mechanism that underlies the failure of this process of separation leading to EA ± TEF is still a matter of debate. Failure of tracheal growth or esophageal atresia has been proposed as primary pathogenic occurrences (38). Regarding the cleavage process itself, various interpretations have been discussed: rapid cranial esophageal growth; rapid caudal esophageal, tracheal, and interposed mesenchymal growth; and rapid growth of the mesenchymal septum interposed between both actively elongating foregut-derived structures and the “folds fusion” theory (39, 40).

Evidence from genetic and toxicologic models (*Noggin* knockout mice and adriamycin-exposed rats, both with up to 70% of tracheoesophageal malformations) implies an abnormal notochord shaping in the failed tracheoesophageal separation (36, 41, 42). In the toxicologic animal model, this early deformed embryonic structure is associated with a disturbed temporospatial pattern of expression of the key developmental gene *Sonic hedgehog* (*Shh*) and members of its signaling cascade suggesting that in the normal development, a precise *Shh* gradient is necessary for separation of the trachea and the esophagus (40, 43).

### The human condition

In isolated EA ± TEF, despite a twinning rate (3.5–5%) significantly higher than in general population (1–2%) and the 1% risk in offspring, the available data do not support a genetic heritability (34). A multifactorial origin with environmental and genetic players is generally accepted. In syndromic EA ± TEF, trisomies 13, 18 or 21 and other chromosomal rearrangements account for 6–10% of cases (44, 45). Although no single specific chromosomal defect has been recognized as an established etiological factor, mutations in *MYCN, CHD7, SOX2, MID1*, and *Gli3* genes were identified in malformative genetic disorders featuring EA ± TEF such as Feingold, CHARGE, AEG, X-linked Opitz, and Pallister–Hall syndromes, respectively (37). These syndromes are not characterized by a disturbed lung phenotype but, interestingly, all these genes involved are expressed during embryonic lung development. *MYCN* (or *N-myc* proto-oncogene that encodes a protein with a basic helix–loop–helix domain) is ubiquitously expressed in early development and at Carnegie stage 15 is differentially and highly expressed in the esophageal and bronchial epithelia. Furthermore, targeted inactivation of the orthologous murine *N-myc* gene revealed its function in the development of the gut and in the branching morphogenesis of the lung, in a way that mutant mice with 25% of wild-type levels of *N-myc* protein are unable to breathe because of severe deficiency in lung branching (46, 47). *CHD7* (chromodomain helicase DNA-binding protein 7) is expressed in organs affected by CHARGE syndrome but is widely expressed during fetal development with high levels in epithelial cells of the lung and gut (48). *SOX2* (member of the SRY-related HMG-box family of transcription factors) is crucial for foregut organogenesis and is transcriptionally expressed during esophageal, tracheal, and lung development. In addition, hypomorphic *SOX2* mutant mice exhibit EA–TEF phenotype and abnormal differentiation of the epithelium lining the conducting airways in the lung (41, 49). *MID1* (a member of the RING-B box-coiled coil subgroup of RING finger proteins) is expressed in lung and esophageal epithelium of human embryos, and experimental data suggest a negative influence in *Shh* expression (50, 51). Finally, *Gli3* is a transcription factor that mediates the *Shh* signaling, a critical pathway in foregut and lung morphogenesis (mutant mice for *Gli3* exhibit tracheoesophageal and lung defects) (40, 52, 53).

### Animal models

Studies on knockout mice demonstrate the involvement of developmental genes such as *Shh, Gli2, Gli3, SOX2, TTF1, Foxf1, RAR* α, and *RAR* β in abnormal tracheoesophageal phenotypes (37, 40).

The spectrum of malformations exhibits varying degrees of severity depending on gene dosage. The esophageal/tracheal phenotype is always accompanied by disturbances of lung development and/or differentiation ranging from agenesis or hypoplasia to lung immaturity or lobulation defects (45). The fact that the abnormal notochord of fetal rats treated *in utero* with adriamycin and bearing EA ± TEF exhibits a disturbed *Shh* gradient also supports the involvement of this gene. *Shh* signaling is a crucial pathway that modulates both digestive and respiratory embryogenesis. In fact, lung hypoplasia with abnormal *Shh* lung signaling and *FGF10* expression in adriamycin-exposed rats with EA–TEF was recently reported [52, 54, Fragoso et al. (submitted to publication)]. In summary, the abovementioned genes plus *BMP* family members implicated in the animal EA–TEF phenotype are key developmental morphogens for both the digestive and respiratory systems modulating esophagus and lung embryogenesis.

## Multifactorial Origin of Respiratory Tract Disease in EA ± TEF Survivors

Respiratory tract malformations or dysfunctions account for a part of the symptoms suffered by EA ± TEF survivors but abnormal function of the gastrointestinal tract, so closely related to the airway, plays a role as well.

### Immaturity and low birth weight

Small for gestational age neonates represent a high-risk group regarding neonatal pulmonary morbidity (55). According to the literature, 20–40% of esophageal atresia babies are born prematurely (<37 weeks) and have low birth weight (56, 57). Deurloo et al. reported that birth weight <2500 g was associated with a complicated clinical evolution during the first year of life although the incidence of preoperative respiratory problems was similar to that of babies born at term (56). Furthermore, Calisti et al. did not find a clear correlation between adverse perinatal conditions (need for ventilatory support at birth, immaturity, or low birth weight) and higher incidence of long-term sequelae, like recurrent respiratory infection and GER in EA ± TEF patients (58). Finally, Legrand et al. reported that neither prematurity nor intrauterine growth retardation was associated with late respiratory symptoms or abnormal spirometry results (3).

### Esophageal dysmotility and GER

The motility of the esophagus is always disturbed in patients with EA ± TEF. Several factors may contribute to this condition like a primary abnormal innervation disorder (59–62) and/or secondary vagal nerve damage during surgical repair (63, 64). Disturbed intrinsic innervation, deficient extrinsic nerve plexus, abnormal neural markers, and altered enteric nerve morphology are also characteristics of the atretic esophagus in the rat and mouse models of EA ± TEF (65–68). These intrinsic anatomic anomalies and/or surgical issues are the main causes for the high prevalence of GER (43%) in these patients (69). Transient lower esophageal sphincter relaxation is the main mechanism underlying GER events in them (70). GER may induce apneic spells or barking cough during infancy, aspiration-related pneumonia, or chronic respiratory disease. However, there is a subset of patients in which the correlation between GER and respiratory symptomatology is not clear. In fact, Soto et al. reported that only 25% of the 70% cases of respiratory distress during the first year of life were GER-related (71). Furthermore, Legrand et al. in a long-term outcome assessment reported a high (75%) prevalence of abnormalities of pulmonary function and respiratory symptoms in survivors of EA ± TEF that were not associated with GER, dysphagia, esophageal dilations, gastrostomy, or fundoplication (3). Gischler et al. reported a similar frequency of respiratory tract infections in refluxing EA ± TEF children who underwent a Nissen fundoplication and those who were treated conservatively, assuming that reflux is not the major contributor for this respiratory pathology (2). Banjar did not find a relationship between GER and the development of chronic lung disease (72).

### Tracheomalacia and other tracheobronchial anomalies

Tracheomalacia is the commonest tracheal defect in EA ± TEF involving up to 78% of patients and is clinically relevant in as many as 10–20% patients (8, 73). The negative intrapleural pressure induced by airway obstruction during respiration seems to be related to GER (74). The varying degrees of severity depend on the extension of the deficient tracheal cartilage and increased length of the transverse muscle in the posterior tracheal wall (30). Tracheomalacia usually is present as barky chronic cough, stridor, and cyanotic episodes in more severe cases. The prevalence seems to decrease with age and its relationship with lower respiratory tract infection or other respiratory symptoms in EA ± TEF survivors is not well established (4).

Congenital tracheal stenosis or web, absence or ectopic right upper bronchus, congenital bronchial stenosis and hypoplastic right upper bronchus, as well as a high incidence of atelectasis have also been described in patients with EA ± TEF (22).

Other abnormal tracheal features in EA ± TEF are impaired mucociliary transport due to the replacement of the ciliated epithelium of the trachea by stratified squamous epithelium (33) and abnormal intrinsic tracheal innervation (31), both likely players in the development and chronic nature of some respiratory symptoms.

Once again, the rodent models of EA ± TEF reproduce nearly identical anomalies. Respiratory tree malformations, disturbed tracheal innervation, and deformed tracheal rings, which determine a smaller lumen and a more flaccid trachea, are invariable in these animals (75–77).

### Lung hypoplasia and other abnormalities

Recent research demonstrated that congenital structural defects of the lung parenchyma itself might also be components of the VACTERL association (28). In fact, a number of reports describe concomitant pulmonary hypoplasia, horseshoe lung, or even lung agenesis (23–29).

Fetal rats with adriamycin-induced EA ± TEF besides showing tracheobronchial malformations similar to humans also present lung hypoplasia and abnormal lung innervation (54, 75, 76). In fact, the abnormal FGF10 and Shh signaling reported in these lungs suggests a disturbed organogenesis with developmental delay [52, Fragoso et al. (submitted to publication)]. Because this rat model closely mimics the EA ± TEF/VACTERL human phenotype reproducing the morphological characteristics of the esophagus and trachea, it suggests that the disturbed lung development described in this model may also occur in humans with EA ± TEF.

## Summary and Conclusion

Respiratory morbidity did not improve and is still highly prevalent in EA ± TEF survivors despite the improvement of perinatal and surgical care, the greater awareness, and more aggressive therapeutic strategies concerning associated morbidity (in particular, GER disease and surgical treatment of tracheomalacia).

Respiratory morbidity associated with EA ± TEF may be due to numerous players intrinsically related to the malformation itself or the surgical treatment (with or without complications), and has been often ascribed to associated disorders like tracheomalacia and GER. Nevertheless, at least in a subset of patients, the epidemiological profile of the respiratory symptoms and its lack of relation to associated conditions suggest other causes or predisposing factors. Because of the scarcity of pathologic material and the difficulties for carrying out embryonic studies in humans, the mutant mice and toxicologic rodent models became invaluable tools in the research of the pathophysiologic processes involved in the disturbed morphogenesis. A fetal origin for the individual predisposition for postnatal lung pathology is supported by the associated tracheobronchial and lung anomalies, the etiological uncertainty regarding several respiratory symptoms, the absence of clear risk factors for poor respiratory outcome, and the unpredictable significance of morbidity in adulthood. Even small disturbances in the process of morphogenesis may impair lung growth and/or maturity. This will be translated into postnatal life as a more or less defective lung growth and/or adaptive response to injury resulting in greater susceptibility to respiratory disturbances. New evidence from experimental models also seems to support this concept because the majority of rodent models featuring the EA–TEF phenotype also display abnormal lung development with different degrees of severity.

In addition to the common embryonic origin of digestive and respiratory systems, human genes identified in syndromic cases of EA ± TEF have been shown to express themselves during lung embryogenesis. These observations allow to hypothesize that their defective expression may also be translated into lung morphogenesis and play some role in subsequent respiratory tract ailments. Both human and experimental research programs are needed to unveil the abnormal mechanisms of lung organogenesis in EA ± TEF in order to answer some of these clinically relevant issues.

## Conflict of Interest Statement

The authors declare that the research was conducted in the absence of any commercial or financial relationships that could be construed as a potential conflict of interest.
